# Polarization-Reconfigurable Metasurface Antenna Design for Drone Terminals Based on Characteristic Mode Analysis

**DOI:** 10.3390/mi17030311

**Published:** 2026-02-28

**Authors:** Shiquan Zhang, Hao Yu, Xianqiong Wen, Hongxing Zheng

**Affiliations:** 1 School of Intelligent Science and Engineering, Xi’an Peihua University, Xi’an 710125, China; wenxianqiong@163.com; 2 School of Electronic Information Engineering, Hebei University of Technology, Tianjin 300401, China; haoyu18702223750@163.com

**Keywords:** metasurface antenna, polarization-reconfigurable, characteristic mode analysis, modal significance, drone terminals

## Abstract

To enhance the anti-jamming performance and operational reliability of drones, this paper presents the design, fabrication, and measurement of a novel polarization-reconfigurable metasurface antenna that meets these demands. The design process is guided systematically by characteristic mode analysis, in which the modal significance coefficient is used as a key tool to predict resonant frequencies and optimize bandwidth. A major innovation lies in the mechanical rotation mechanism, which enables the antenna to switch between left-hand circular polarization, linear polarization, and right-hand circular polarization, thereby avoiding losses associated with active electronic components. The antenna features a compact geometry of 0.49λ × 0.49λ and delivers strong performance across all polarization states. Impedance bandwidth exceeds 29.9%, average gain ranges from 5.1 to 6.0 dBi, and high polarization purity is achieved with an axial ratio bandwidth > 10% in circular polarization modes and cross-polarization discrimination >23 dB in the linear polarization state. Simulated and measured results are in good agreement, confirming the effectiveness and robustness of the proposed design for modern sub-6 GHz 5G drone terminals.

## 1. Introduction

**Figure 1:**
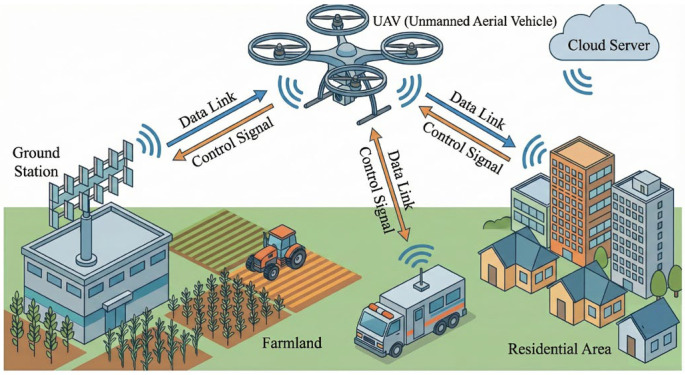
Complex application scenario of the UAV with control signals coexisting with interference (generated by the AI-assisted method).

With the expanding application of unmanned aerial vehicles (UAVs) or drones, the operational reliability of their communication links, particularly anti-jamming capability, has become increasingly critical. Leveraging the polarization characteristics of electromagnetic waves presents a significant technical approach to address such challenges. The polarization properties of antennas, especially the ability to dynamically reconfigure polarization in response to varying operational scenarios—known as polarization reconfigurability—constitute a key research focus in this field. The application scenario is illustrated in Figure 1. The design targets the sub-6 GHz spectrum, crucial for beyond-visual-line-of-sight (BVLOS) drone communications, where the antenna’s compact size (0.49λ × 0.49λ), lightweight structure, and polarization agility directly address payload and aerodynamic integration constraints while enhancing link reliability against interference.

The rapid deployment of 5G and the ongoing evolution towards 6G technology impose increasingly stringent requirements on terminal antennas, compelling a focused research effort on achieving miniaturization, wide impedance bandwidth, high gain, and adaptive functionality within extremely confined spaces. The limited physical size of modern mobile devices necessitates antenna designs that are not only compact but also capable of supporting high data rates and reliable connectivity. Traditional microstrip antennas, while low-profile and easy to integrate, fundamentally struggle to meet these multifaceted demands due to their inherent limitations in bandwidth, gain, and functional agility [1].

Metasurface antennas, which leverage two-dimensional artificial electromagnetic structures composed of subwavelength unit cells, have emerged as a profoundly promising alternative [2]. They offer a unique combination of a low profile, high integration potential, and exceptional control over wavefronts and polarization states, enabling performance characteristics that are difficult to achieve with conventional designs. Significant research efforts have been directed towards addressing three critical challenges in antenna technology for modern wireless systems [3,4]. The first is miniaturization, which is paramount for integration into compact smart terminals and Internet of Things (IoT) devices. Techniques such as employing slotting and branch-loading on patches, as well as incorporating defected ground structures (DGS) [5], have been effectively used to reduce antenna size and achieve multi-band operation. The second key area is the generation of circular polarization, which is highly desirable for its robustness against multipath fading and orientation mismatches between transmitting and receiving antennas [6]. It has been proven that metasurfaces are exceptionally capable in this regard. It was often used as a superstrate to transform a linearly polarized source into a radiator with a wide axial ratio bandwidth [7]. The critical research focus is reconfigurability [8], which allows a single antenna to dynamically adapt to varying channel conditions and communication standards.

Polarization configurability, in particular, is crucial for optimizing signal quality, mitigating interference, and improving compatibility with diverse network infrastructures. Conventional reconfiguration techniques predominantly rely on active components like PIN diodes or varactors [9]. However, these introduce well-documented drawbacks, including insertion loss, design complexity, DC power consumption, and the potential for harmonic radiation [10].

To overcome these limitations, a promising direction is the development of passive, mechanically reconfigurable antennas. The design process for such innovative antennas can be rigorously guided by characteristic mode analysis (CMA) [11], a powerful computational method for analyzing the inherent resonant properties (or characteristic modes) of a conducting structure without the influence of a specific excitation. The modal significance (MS) parameter serves as a central design metric within CMA [12], quantitatively identifying the most significant resonant modes of the metasurface structure. This analytical approach provides deep physical insight and systematically guides the structural evolution towards optimal performance for both miniaturization and multi-polarization generation [13]. Research has demonstrated that CMA can be effectively used to design metasurfaces that excite multiple broadside modes, which is essential for achieving a wide impedance bandwidth.

Polarization-reconfigurable antennas represent an advanced form of antenna technology. They can dynamically switch or adjust their polarization states—such as linear or circular polarization—based on the channel environment and interference conditions. This capability allows the antenna to maintain optimal matching with the signal while maximizing interference suppression, which requires intelligent sensing and fast control algorithms.

While the CMA MS are well-established tools in antenna engineering, the primary innovation of this work lies in their systematic and targeted application to co-design a miniaturized metasurface unit cell and a passive mechanical rotation mechanism. This integrated approach achieves three distinct polarization states within an exceptionally compact footprint of 0.49λ × 0.49λ, specifically optimized for drone terminal constraints. CMA was not merely used for analysis but actively guided the structural evolution—from a simple square patch to the final triple-hollow unit—by leveraging MS to predict and lower resonant frequencies, separate orthogonal modes, and ultimately optimize for wide axial ratio bandwidth and high polarization purity across all reconfigured states.

In this approach, the principal contributions of this paper are as follows.

**(i) Systematic Design via CMA**

The application of CMA and MS analysis to systematically design and optimize a miniaturized metasurface unit cell. This method enables a targeted adjustment of the structure’s resonant properties, achieving a significant reduction in operational frequency and ensuring optimal excitation of desired modes for wideband performance.

**(ii) Low-Loss Polarization Switching**

The introduction of a simple yet effective mechanical rotation mechanism applied to the metasurface layer. This passive approach enables low-loss and highly reliable polarization switching among left-hand circular polarization (LHCP), linear polarization (LP), and right-hand circular polarization (RHCP) states, effectively eliminating the losses and complexities associated with active electronic components.

**(iii) Experimental Validation of a High-Performance Antenna**

The realization and experimental validation of a compact antenna prototype that demonstrates a wide impedance bandwidth (>29.9%), stable gain across all polarization states (5.1 to 6.0 dBi), and high polarization purity. The excellent agreement between simulation and measurement confirms its potential as a robust solution for 5G terminal devices.

Polarization reconfigurability serves as an effective electronic counter-countermeasure technique. For instance, a cross-polarization discrimination (XPD) level greater than 20 dB, as achieved by this design, can improve the signal-to-interference ratio (SIR) by a similar magnitude when facing a cross-polarized jammer. Furthermore, the ability to dynamically switch between LHCP and RHCP can circumvent jamming strategies that target a specific polarization, thereby significantly enhancing the robustness and anti-jamming margin of the drone’s communication link.

This approach aims to bridge the gap between the demanding requirements of modern wireless communication and the limitations of current antenna solutions by presenting a passively reconfigurable metasurface antenna that excels in size, bandwidth, and adaptive functionality. To achieve the above, the paper is structured as follows. Section 2 introduces the design principles, including the theoretical basis of characteristic modes and MS analysis. Section 3 presents simulation results. To verify our newly designed antenna, experimental results and analysis are provided in Section 4. Finally, we offer conclusions in the last section.

## 2. Antenna Design Principles with MS Analysis

### 2.1. Theoretical Foundation of Characteristic Modes

The theory of characteristic modes provides a deterministic framework for analyzing the modal behavior of a conducting body. It involves solving a weighted eigenvalue equation derived from the method of moments impedance matrix [14].

*XJ_n_* = *λ_n_RJ_n_* (1)

where *J_n_* is the *n*-th mode characteristic current, rand *X* is the real and imaginary parts of the impedance matrix, and *λ_n_* is the associated eigenvalue. A key derived parameter is the *MS* defined by

*MS*_n_ = 1/|1 + j*λ*_n_| (2)

The *MS* indicates how easily a mode can be excited. Its value close to one signifies a strong, easily excited mode that dominates the radiation at a specific frequency. Since CMA is independent of the feed, it offers profound physical insight into the structure’s intrinsic resonances, making it ideal for initial design and optimization.

The CMA was performed using the integral equation solver within CST Microwave Studio. To analyze the inherent modes of the metasurface unit cell, periodic boundary conditions were applied to simulate an infinite array environment during the initial design phase. The conductor was modeled as a perfect electric conductor (PEC). An adaptive meshing scheme was employed, ensuring a minimum of 20 cells per wavelength at the highest frequency of interest (≈6 GHz). The first 10 characteristic modes were calculated, with modes 1–4 identified as the most significant for the operating bands. The final antenna performance, including feed interaction, was validated using a finite-array model.

### 2.2. CMA-Guided Evolution of the Metasurface Unit Cell

**Figure 2:**
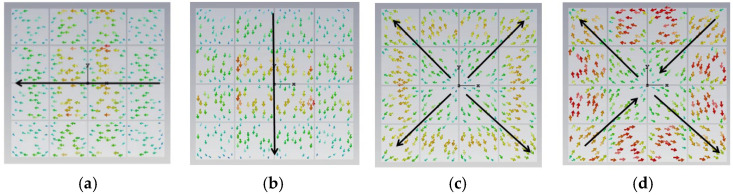
Surface current distribution of periodic square metasurface for the 4 × 4 array: (**a**) mode 1, (**b**) mode 2, (**c**) mode 3, and (**d**) mode 4. Modes 3 and 4 especially show orthogonal current distribution.

**Figure 3:**
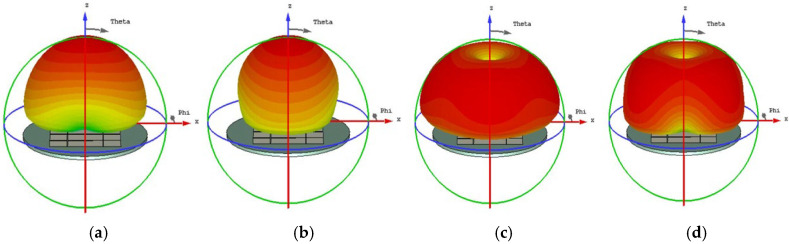
Radiation pattern of a square unit metasurface for the 4 × 4 array: (**a**) mode 1, (**b**) mode 2, (**c**) mode 3, and (**d**) mode 4. Modes 1 and 2 show almost the same pattern, as well as modes 3 and 4.

**Figure 4:**
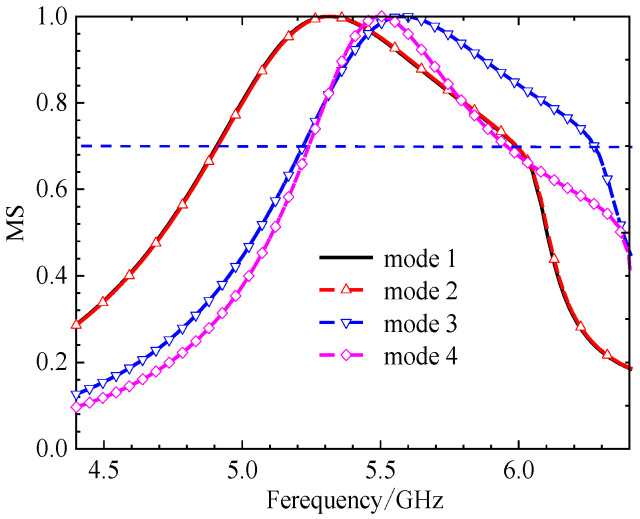
The MS of a 4 × 4 square unit metasurface. Modes 1 and 2 show almost the same MS distribution, modes 3 and 4 are different; the MS peaks are narrow.

**Figure 5:**
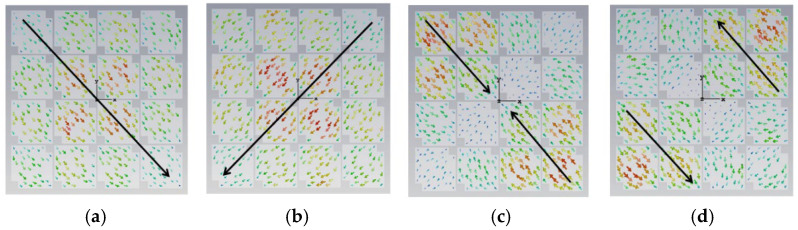
Current distribution on the 4 × 4 metasurface while each square unit cut off a pair of opposite corners, in cases of (**a**) mode 1, (**b**) mode 2, (**c**) mode 3, and (**d**) mode 4.

**Figure 6:**
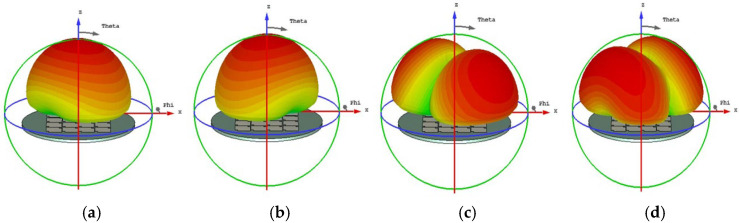
Radiation pattern of 4 × 4 metasurface while each square unit cut off a pair of opposite corners, (**a**) mode 1, (**b**) mode 2, (**c**) mode 3, and (**d**) mode 4.

**Figure 7:**
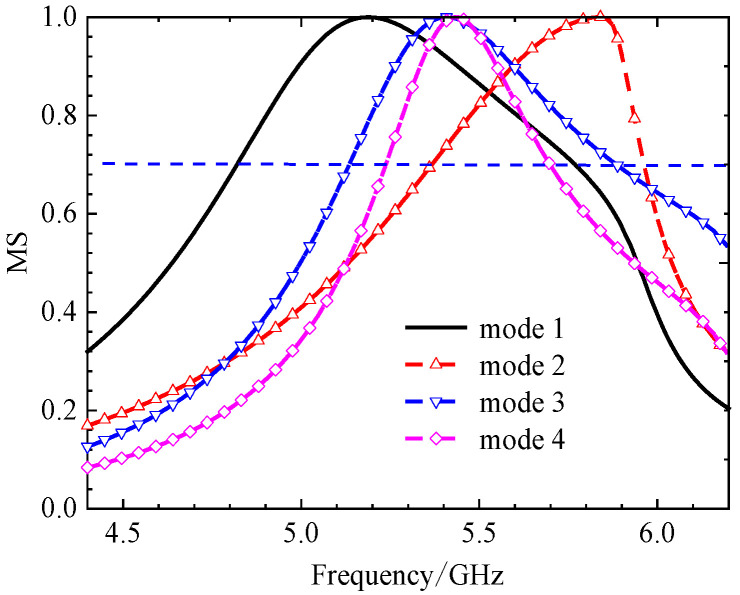
MS of the 4 × 4 unit metasurface, with each square unit cut off a pair of opposite corners. The peaks shifted to a lower frequency.

**Figure 8:**
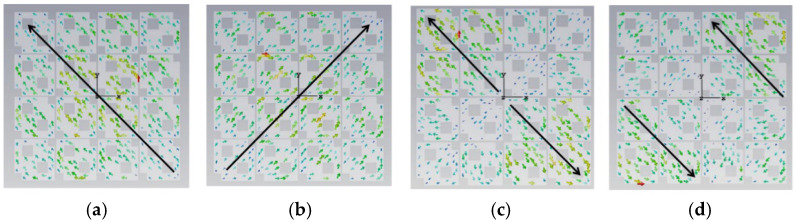
Surface current distribution of each unit with two hollows (small squares): (**a**) mode 1, (**b**) mode 2, (**c**) mode 3, and (**d**) mode 4.

**Figure 9:**
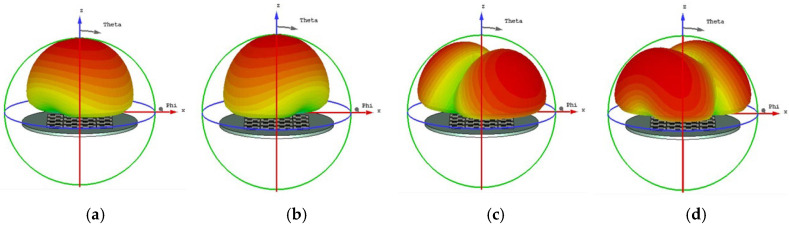
Radiation pattern of each unit with two hollows (small squares): (**a**) mode 1, (**b**) mode 2, (**c**) mode 3, and (**d**) mode 4.

**Figure 10:**
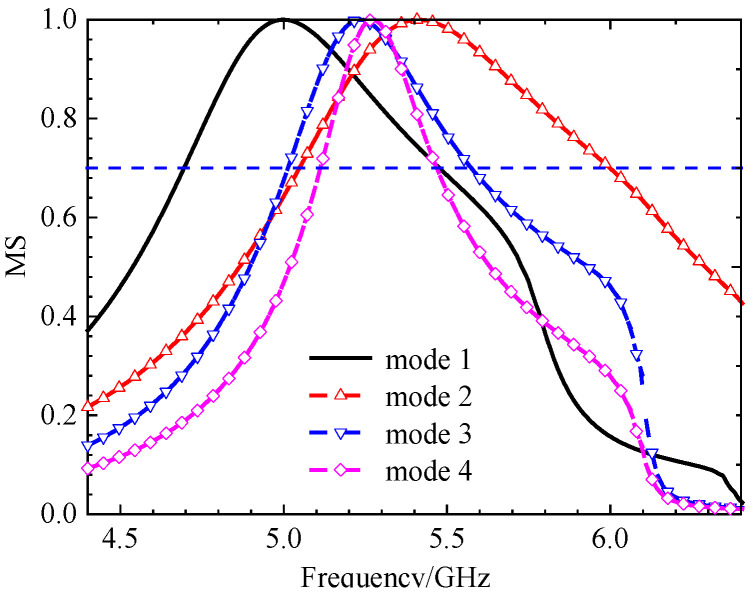
MS of each unit with two small hollow squares; peak frequencies moved lower, but modes 3 and 4 are too closely spaced.

**Figure 11:**
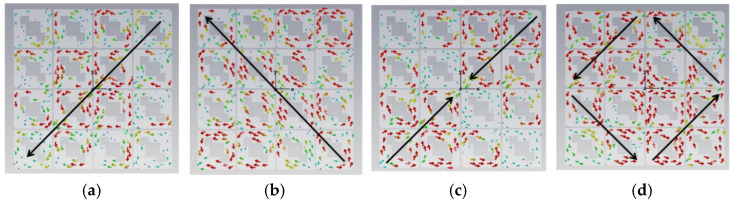
Surface current distribution of a small square with three hollows: (**a**) mode 1, (**b**) mode 2, (**c**) mode 3, and (**d**) mode 4.

**Figure 12:**
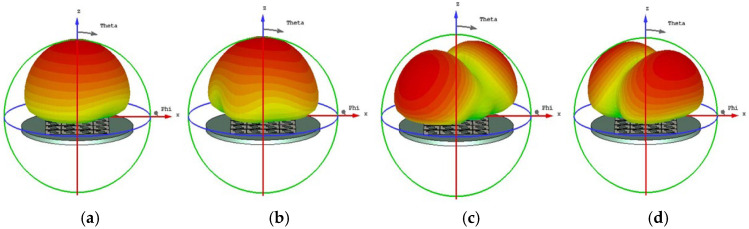
Radiation pattern of 4 × 4 metasurfaces, each square unit with three hollow small squares: (**a**) mode 1, (**b**) mode 2, (**c**) mode 3, and (**d**) mode 4.

**Figure 13:**
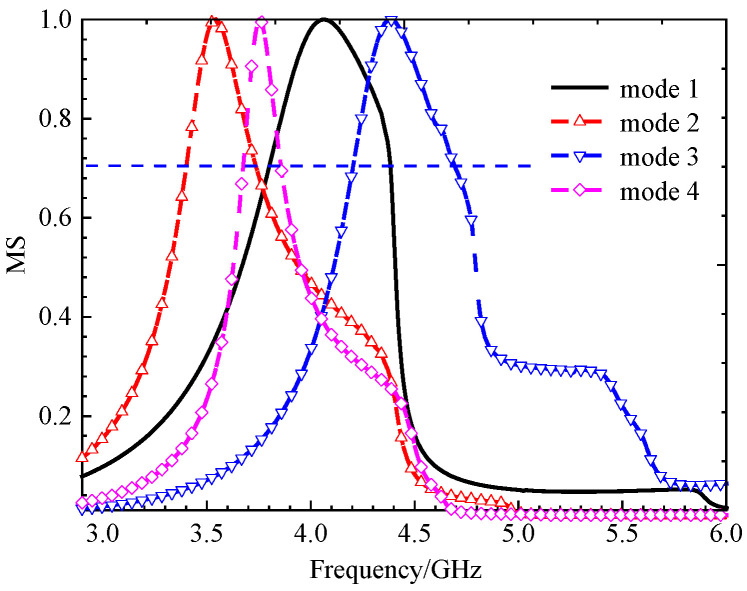
MS of 4 × 4 metasurfaces, each unit with three hollow small squares. The peak frequency has enough space.

Without generality, the core of the antenna consists of a 4 × 4 periodic metasurface array. In order to present the metasurface as an antenna’s radiator with a polarization transformer, we verify the unit cell changing through four stages, and the CMA method is used. By using CST Microwave Studio’s integral equation solver, the evolution of the surface current distribution and MS has been checked. We summarize four stages, as shown in Figure 2, Figure 3, Figure 4, Figure 5, Figure 6, Figure 7, Figure 8, Figure 9, Figure 10, Figure 11, Figure 12 and Figure 13. These figures not only indicate the law of polarization changes with antenna structure changes but also guide our design.

**(i) Square Patch**

The initial analysis of a simple square patch revealed weak current distributions for the four modes, as shown in Figure 2. While modes 3 and 4 showed orthogonal currents, their radiation pattern for these modes were depicted in Figure 3. We found these patterns in almost the same form. The MS peaks were narrow and occurred at a high frequency of approximately 7 GHz, as shown in Figure 4; it is unsuitable for a compact design.

**(ii) Patch with Corner Cutouts**

When cutting off a pair of opposite corners of a square [15], we can enhance the orthogonal current components. The current distributions and radiation patterns of the four models are depicted in Figure 5 and Figure 6, respectively. The MS peaks moved to lower frequency, to about 5.18 GHz (mode 1) and 5.83 GHz (mode 2) with wider bandwidths. However, degenerate modes (modes 3 and 4) posed a risk of mutual interference, as shown in Figure 7.

**(iii) Patch with Two Hollows**

In order to reduce the size, the frequency must be moved to a lower value. Therefore, we can consider improving the structure of the metasurface unit. Based on step 2, if we add two symmetric hollows in the metasurface unit, it will further increase the current path length. Current distributions and radiation patterns are illustrated in Figure 8 and Figure 9, respectively. Then, the MS of these four modes is plotted in Figure 10. We can see that peaks of modes 1 and 2 are reduced to 4.98 GHz and 5.41 GHz, respectively. However, the modes remained too closely spaced for other modes. It must be improved further.

**(iv) Final Design with Three Hollows**

From Figure 10, we found that for MS = 1, the bandwidth is not enough. Incorporating a third interconnected hollow significantly intensified the current density and elongated the path. We can improve the structure of the unit. The final optimization yielded well-separated, strong MS peaks at 4.06 GHz (mode 1), 3.56 GHz (mode 2), 4.38 GHz (mode 3), and 3.76 GHz (mode 4). The corresponding surface current distributions and radiation patterns, depicted in Figure 11 and Figure 12, show distinct and orthogonal modes suitable for generating circular polarization.

Parametric studies based on MS were conducted to finalize key dimensions, such as the unit cell side length *w*_0_ and the hollow size *w*, leading to the optimal values of *w*_0_ = 11.0 mm and *w* = 3.68 mm. The same method leads to the final dimensions *w*_1_ = 3.68 mm. More importantly, the characteristic angle difference between the orthogonal modes 1 and 2 was approximately 90° within the 3.62–4.25 GHz band, confirming their potential to generate circular polarization when excited with equal amplitude and a 90° phase difference. This property will be summarized in the next section. With three hollow small squares, the MS of metasurfaces is depicted in Figure 13. The procedure for finding the size will be discussed below.

## 3. Overall Antenna Structure and Reconfiguration Mechanism

**Figure 14:**
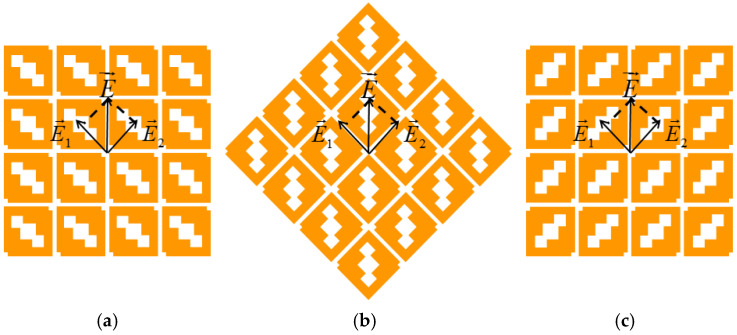
Schematic diagram of the polarization-reconfigurable metasurface: (**a**) initial position, (**b**) 45° rotation, and (**c**) 90° rotation.

From the analysis above, we can implement the polarization reconfiguration of the *metasurface* antenna. Its principle, as shown in Figure 14, can be concluded as follows.

**(i) 0° Position (LHCP).** The metasurface orientation decomposes the incident field into orthogonal components (*E*_1_, *E*_2_). The asymmetric loading (hollows) introduces a 90° phase lead in *E*_1_ relative to *E*_2_, generating LHCP.

**(ii) 45° Rotation (LP).** Rotating the metasurface by 45° equalizes the path lengths and phase for the orthogonal components, resulting in LP.

**(iii) 90° Rotation (RHCP).** Further rotation to 90° reverses the phase relationship, causing *E*_1_ to lag *E*_2_ by 90°, generating RHCP.

When the metasurface is in its initial position, as shown in Figure 14a, the vector electric field E parallel to the x-axis is decomposed into two orthogonal components, *E*_1_ and *E*_2_. The amplitudes of these two components are equal. Since squares are etched on the metasurface, the two vector components travel paths of different electrical lengths, resulting in *E*_1_ leading *E*_2_ by a phase difference of 90 degrees, thus achieving the LHCP. When the metasurface structure is rotated by 45°, as shown in Figure 14b, the amplitudes and phases of *E*_1_ and *E*_2_ become equal, and the antenna realizes a conversion to linear polarization. When the metasurface structure is rotated by 90°, as shown in Figure 14c, *E*_1_ lags *E*_2_ by a phase difference of 90°, and the antenna achieves conversion from LP to RHCP.

### 3.1. Implementation of Polarization Reconfiguration

**Figure 15:**
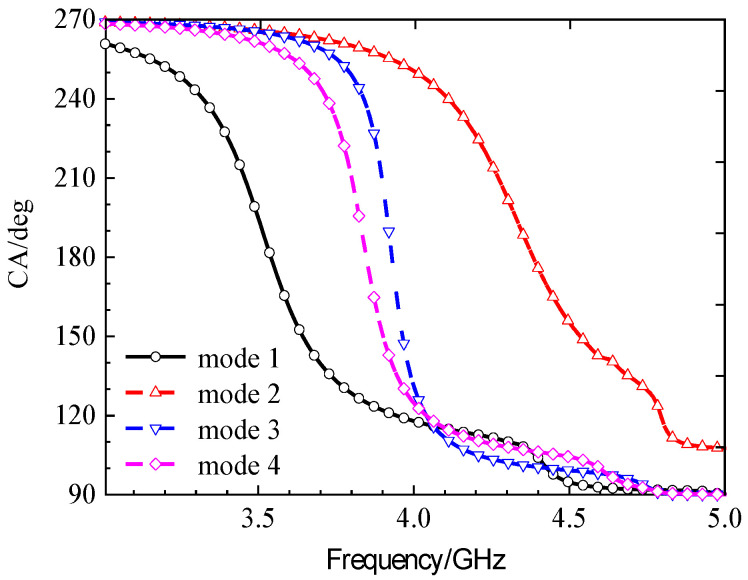
Characteristic angle (CA) of metasurfaces with three hollow small squares.

The characteristic angles are shown in Figure 15. Within the frequency band of 3.62–4.25 GHz, the characteristic angles of mode 1 and mode 2 differ by approximately 90°. Combining the analysis of the surface currents and radiation patterns for each mode presented above, the antenna can achieve LHCP in its initial position. Furthermore, through mechanical rotation, it can achieve polarization conversion from LHCP to LP and then to RHCP.

Having determined the structure and specific parameter values of the metasurface unit, the feasibility of achieving circular polarization with the metasurface is next analyzed based on the characteristic angles obtained from characteristic mode theory. The characteristic angle diagram of the metasurface is shown in Figure 15. From 4.78 GHz to 4.98 GHz, there is an approximate 90-degree phase difference between mode 1 and mode 2. Combined with the 90-degree angular difference in the surface current flow directions of mode 1 and mode 2 shown in Figure 11, and the identical radiation directions of mode 1 and mode 2 shown in Figure 12, when a suitable excitation source is designed to excite these two modes of the metasurface, the conditions for achieving circular polarization are met because the amplitudes of the two modes are equal, the current flow directions differ by 90 degrees, and their phases differ by 90 degrees. Therefore, the antenna can exhibit circular polarization characteristics within this frequency band.

The design progression was governed by quantitative targets derived from CMA. A mode was considered strongly excitable and suitable for efficient radiation if its MS exceeded 0.7. Furthermore, to generate high-purity circular polarization, the characteristic angle difference between the two targeted orthogonal modes (Modes 1 and 2) was required to be 90° ± 15° over the target frequency band. The evolution from the two-hollow to the three-hollow structure was decisive, as it successfully increased the MS of the fundamental modes while achieving the necessary ~90° characteristic angle separation across the 3.62–4.25 GHz band (see Figure 15).

**Figure 16:**
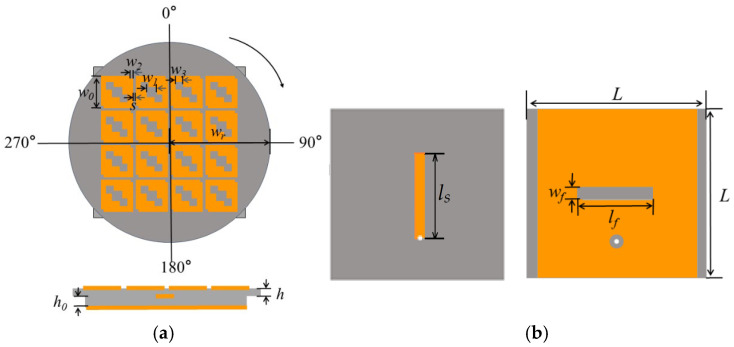
Final designed antenna structure, metasurfaces with three hollow small squares: (**a**) top and side view, (**b**) bottom view (feed and ground).

The complete antenna structure, illustrated in Figure 16, comprises two FR4 dielectric substrates (*ε*_r_ = 4.4, tan*δ* = 0.02). Top Layer: A circular substrate carrying the optimized 4 × 4 units forming a metasurface. Bottom Layer: A square substrate featuring a slot-coupled feeding structure on its top side and a partial ground plane with a coupling slot on the bottom. A 50 Ω coaxial probe feeds the antenna from underneath. This is a simple yet practical method for the low-profile antenna design of unmanned aerial vehicles (UAVs), as shown in Figure 1 [16]. The final size of the designed antenna is listed in Table 1 below.

A slot-coupled feed provides stable excitation across configurations. Key feed parameters (slot length *l_f_*, width *w_f_*, stub length *ls*, and the same width as the slot) were optimized via parametric studies in CST for impedance matching across all states. Polarization reconfiguration is achieved by mechanically rotating the top circular metasurface layer relative to the fixed bottom feed layer. The principle is illustrated as follows.

At 0° (LHCP State), the asymmetrical loading (three hollows) of the metasurface causes the incident field to decompose into two orthogonal components (*E*_1_ and *E*_2_). The structure introduces a 90° phase lead for *E*_1_ relative to *E*_2_, resulting in LHCP radiation.

At 45° (LP State), rotating the metasurface by 45° equalizes the path lengths and phase for the orthogonal components, leading to linear polarization.

At 90° (RHCP State), at this orientation, the phase relationship is reversed, causing *E*_1_ to lag *E*_2_ by 90°, thus generating RHCP.

Key parameters of the feeding structure (slot length *lf*, width *wf*, and stub length *ls*) were optimized via parametric studies in CST to ensure good impedance matching across all three polarization states.

The slot-coupled feed structure was optimized not only for impedance matching but also to efficiently excite the targeted orthogonal characteristic modes (Modes 1 and 2). The position and length (*lf*) of the coupling slot were iteratively adjusted to maximize the coupling to the surface current distributions of these modes, as shown in Figure 11, ensuring the excitation of two orthogonal modes with equal amplitude and the required 90° phase difference for high-quality circular polarization.

**Figure 17:**
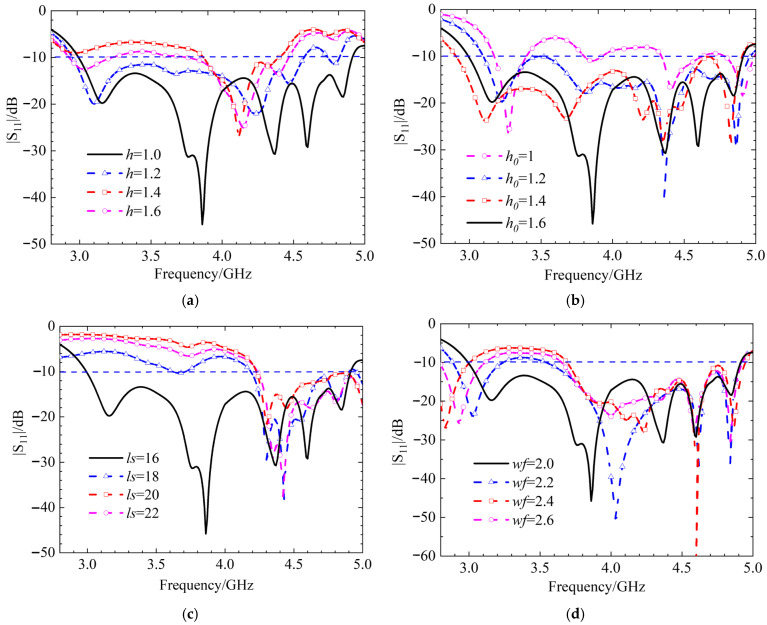
Simulation results of |S_11_| for different thicknesses of circular dielectric substrates *h*, square dielectric substrates *h*_0_, strip patch length *ls*, and gap width *wf*, (**a**) parameters *h*, (**b**) parameters *h*_0_, (**c**) parameters *ls*, and (**d**) parameters *wf*.

**Figure 18:**
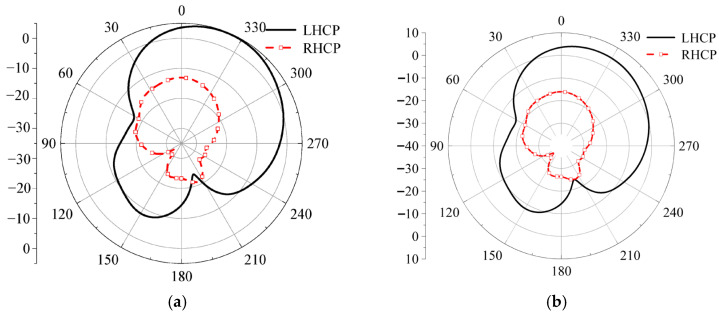
Simulation diagram of LHCP and LHCP, (**a**) phi = 0°, (**b**) phi = 90°.

Next, we will check the influence of various antenna structures and parameters on the S-parameters under three polarization states. First, the initial position, i.e., the left-hand circular polarization state, is discussed. The effects of the circular dielectric substrate thickness *h*, the square dielectric substrate thickness *h*_0_, the strip patch length *ls*, and the slit width *wf* on the S-parameters are analyzed, and the simulation results are shown in Figure 17. When the thicknesses of the two dielectric substrates are altered, the trend of the antenna’s |*S*_11_| simulation results remains essentially unchanged. Only when the thickness changes significantly does the reflection coefficient of the antenna increase due to impedance mismatch, leading to a substantial reduction in the impedance bandwidth. As the length of the strip patch and the width of the slit increase, the excited modes change, causing the resonant frequency to shift towards higher frequencies and the impedance bandwidth to decrease. The radiation patterns for LHCP and RHCP are shown in Figure 18. In this case, the LHCP is about 18 dBi higher than the RHCP, indicating that the antenna achieves the characteristics of LHCP.

**Figure 19:**
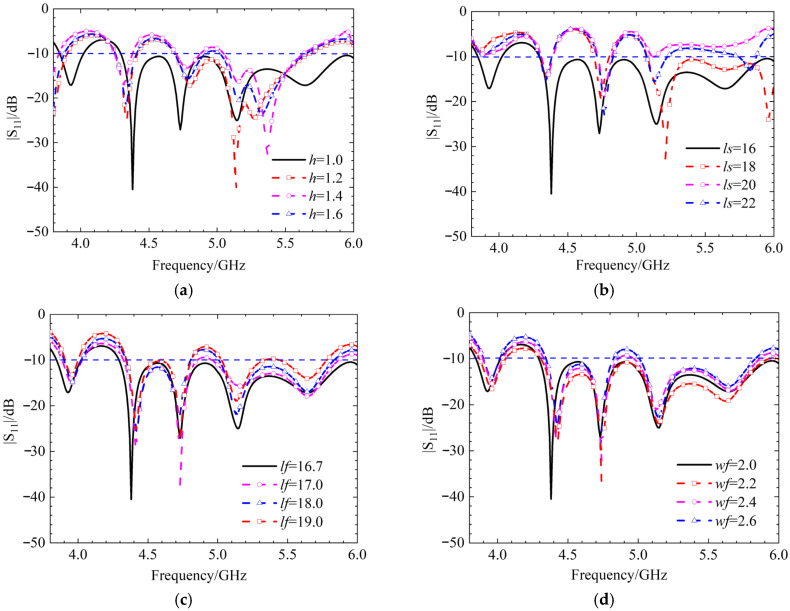
Simulation results of |S_11_| for different thicknesses of circular dielectric substrates *h*, strip patch length *ls*, gap length *lf*, and gap width *wf*, (**a**) parameters *h*, (**b**) parameters *ls*, (**c**) parameters *lf*, and (**d**) parameters *wf*.

Continuing the discussion on the metasurface rotated at 45° in the linear polarization state, the influence of the numerical values of the circular dielectric substrate thickness *h*, the length of the strip patch *ls*, the gap length *lf*, and the gap width *wf* on the S-parameters is analyzed, with simulation results shown in Figure 19. When the thickness of the circular dielectric substrate changes, the variation in the simulated |S_11_| of the antenna is minimal, with only certain frequency bands developing stopbands, indicating a degree of stability. When the length of the strip patch increases, the impedance mismatch becomes more severe, preventing the antenna from functioning properly.

**Figure 20:**
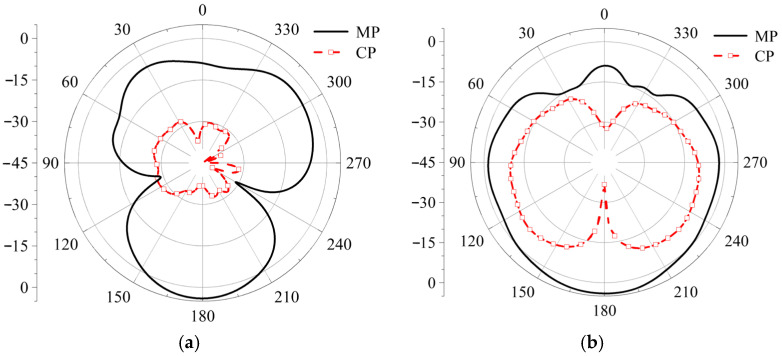
Simulation diagram of main-polarization (MP) and cross-polarization (CP), (**a**) phi = 0°, (**b**) phi = 90°.

When the length and width of the gap are altered, the S-parameters of the antenna remain almost unchanged, demonstrating strong stability in the coupling feed structure. The radiation patterns for the co-polarization and cross-polarization are shown in Figure 20. At both phi = 0° and phi = 90°, the co-polarization level is approximately 20 dBi higher than the cross-polarization level, indicating that the antenna exhibits good anti-interference characteristics [17].

The following analysis delves into the final optimized configuration of the metasurface-based antenna, specifically when it is rotated by 90 degrees to operate in the RHCP state. A detailed parametric study is crucial for understanding how different physical dimensions influence the antenna’s performance, particularly its *S*_11_.

The study investigates the impact of four key parameters, circular dielectric substrate thickness *h*, square dielectric patch thickness *h*_0_, coupling gap length *lf*, and coupling gap width *wf*.

**Figure 21:**
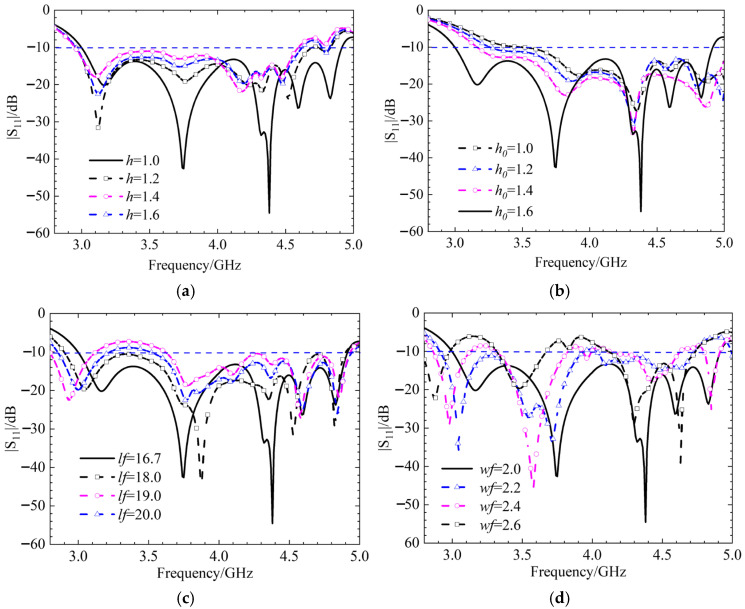
Simulation results of |S_11_| for different thicknesses of circular dielectric substrates *h*, square dielectric substrates *h*_0_, gap length *lf*, and gap width *wf*, (**a**) parameters *h*, (**b**) parameters *h*_0_, (**c**) parameters *lf*, and (**d**) parameters *wf*.

The findings, summarized in Figure 21, reveal distinct behaviors for each parameter.

When the thicknesses of the dielectric substrates, *h* and *h*_0_, are altered, the antenna’s impedance bandwidth experiences a slight decrease. However, a key indicator of stability is that the resonant frequency points remain almost unchanged. This suggests that while the range of frequencies over which the antenna is well-matched to the feed line narrows slightly, the fundamental operating frequencies are robust against variations in substrate thickness. This stability is often a desirable characteristic in antenna design, as shown in Figure 21a,b.

The analysis shows that the length of the gap, *lf*, in the coupling structure has only a minor impact on the antenna’s overall performance. This implies that the design is not highly sensitive to minor manufacturing tolerances in this specific dimension, as shown in Figure 21c.

In contrast to the gap length, the width of the gap, *wf*, has a significant and critical influence [18]. As the gap width increases, a notable phenomenon occurs: the entire operating frequency band shifts towards lower frequencies. Furthermore, undesired stopbands (frequency bands where signal transmission is blocked) begin to appear within the intended operating band. This can severely degrade antenna performance and is a critical consideration during the design and tuning process, as shown in Figure 21d.

**Figure 22:**
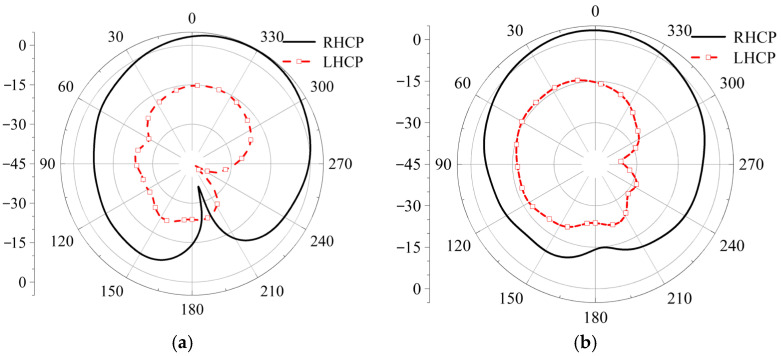
Simulation diagram of LHCP and RHCP, (**a**) phi = 0°, (**b**) phi = 90°.

The radiation patterns for both left-hand circular polarization (LHCP) and right-hand circular polarization (RHCP) are presented in Figure 22. The results provide clear and compelling evidence for the antenna’s polarization state. At two principal planes, phi = 0° and phi = 90°, the RHCP gain is approximately 21 dBi higher than the LHCP gain in the direction of maximum radiation.

This substantial difference, known as the axial ratio performance in practice, serves as a definitive confirmation that the antenna successfully achieves high-purity right-hand circular polarization. The 90-degree rotation of the metasurface effectively excites the desired orthogonal modes with a 90-degree phase difference, which is the fundamental principle for generating circular polarization, while significantly suppressing the unwanted LHCP component.

### 3.2. Practical Considerations for Mechanical Reconfiguration

The proposed mechanical rotation mechanism offers significant advantages in terms of low insertion loss and high reliability by circumventing active electronic components. However, several practical aspects warrant discussion for system-level implementation.

For integration into a UAV terminal, the top metasurface layer can be mounted on a shaft connected to a **micro-stepper motor** equipped with an optical encoder for precise angular control. The entire assembly should be housed within a low-profile, aerodynamic radome to protect against environmental factors (moisture, dust) and minimize vibration or airflow-induced perturbations during flight. The estimated total added mass for the rotation mechanism is minimal, adhering to typical drone payload constraints.

**Switching Speed and Application Scope:** The mechanical switching time is estimated to be on the order of milliseconds (e.g., ~200 ms for a 45° step), governed by the inertia of the rotating layer and the micro-stepper motor’s performance. This speed is sufficient for mission-based or environment-adaptive polarization switching but is not intended for real-time, symbol-rate adaptive beamforming. Typical use cases for a drone terminal include reconfiguring the polarization during different mission phases (e.g., takeoff/landing vs. cruise) or in response to identified persistent interference sources, where switching latency at the millisecond level is acceptable.

**Angular Precision and Feedback:** To maintain the high polarization purity demonstrated in measurements (e.g., XPD > 23 dB), precise angular control is necessary. In an open-loop configuration, cumulative mechanical errors could degrade performance. For practical deployment, we recommend the integration of a closed-loop feedback system, such as an optical or magnetic encoder, with the driving motor to ensure accurate angular positioning (e.g., within ±2°).

**Power Consumption Consideration:** While the rotating mechanism itself is passive, the actuating motor requires power during the switching transient. A key advantage over active (e.g., PIN diode) methods is that our approach consumes zero dc power in a static state. For applications where polarization changes are infrequent (low duty cycle), the average power consumption of the mechanical system can be significantly lower than that of continuously biased active components. A trade-off analysis between switching speed, precision, and average power should be conducted based on specific application requirements.

In summary, the parametric study confirms the stable operation of the antenna’s core resonance while highlighting the gap width as a critical tuning parameter. The radiation pattern measurements ultimately validate the design’s success in achieving excellent RHCP performance. We can conclude the final size of the designed antenna as in Table 1. According to these sizes, the antenna can be fabricated and then tested. Results will be discussed next section. The key performance indicators are as follows, and all of them meet the design requirements.

For **bandwidth**: The impedance bandwidth, defined as the −10 dB reflection coefficient (|S_11_|) bandwidth, exceeds 48%, 32%, and 48% in the case of LHCP, LP, and RHCP, respectively.

For **gain**: The **avg. gain** remains stable, bigger than 5 dBi in three polarization cases.

For circular polarization: The antenna exhibits a 3 dB axial ratio (AR) bandwidth of 9%.

For linear polarization: The linear polarization demonstrates a high cross-polarization discrimination (XPD) level of >23 dB, defined as the ratio of co-polarization to cross-polarization gain.

## 4. Experimental Results

### 4.1. Fabrication and Measurement Setup

**Figure 23:**
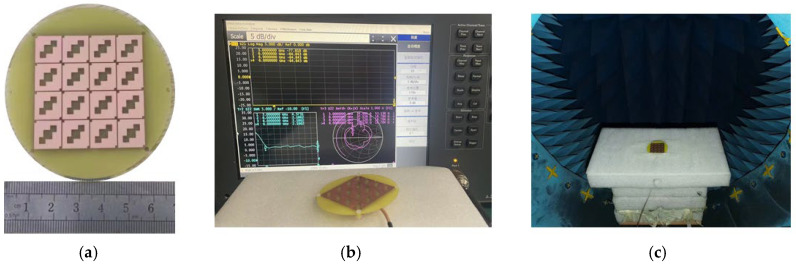
Physical and test images of antennas: (**a**) physical image of antenna, (**b**) test diagram of antenna connection to vector network, and (**c**) test diagram of line in microwave anechoic chamber.

After the overall structure of the reconfigurable metasurface antenna was determined through simulation and analysis, the antenna was fabricated, and its parameters were measured. A prototype antenna was fabricated using a precision printing and metallization process on FR4 substrate. The photograph of the fabricated prototype and the measurement setup are shown in Figure 23. The measured performance was characterized using a vector network analyzer (Keysight N5224A) for S-parameters and in an anechoic chamber for radiation patterns, gain, and axial ratio.

**Figure 24:**
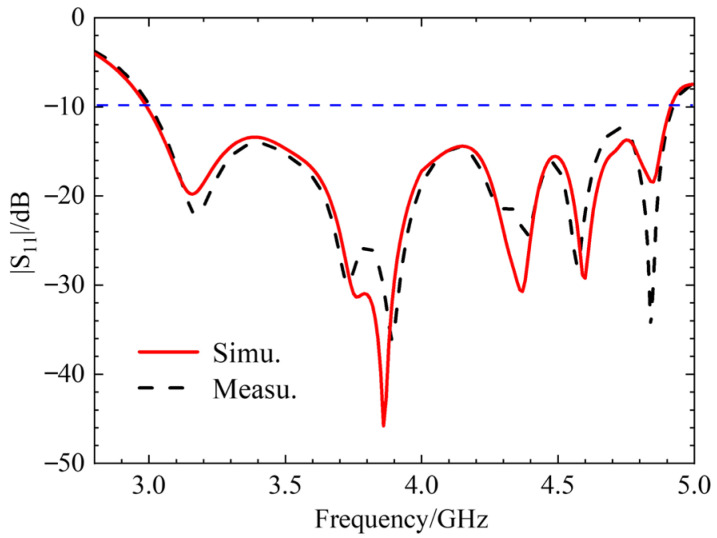
Comparison between simulation (Simu.) and measurement (Measu.) of |*S*_11_| antenna at initial position.

Figure 23a shows a photograph of the fabricated antenna prototype. Figure 23b,c depict the antenna connected to a vector network analyzer for measurement and the antenna under test in an anechoic chamber, respectively. The antenna was first connected to the analyzer. Based on the voltage standing wave ratio (VSWR) and the Smith chart, the antenna was adjusted, and its |*S*_11_| parameter was measured, as shown in Figure 24.

**Figure 25:**
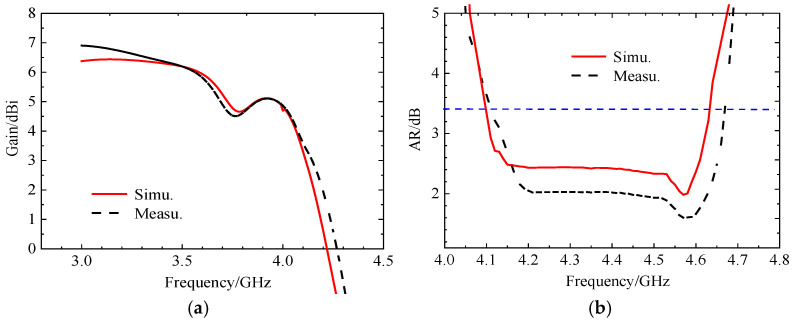
Comparison between simulation and testing of gain and axial bandwidth at initial position: (**a**) gain chart, (**b**) axis ratio (AR) bandwidth graph.

**Figure 26:**
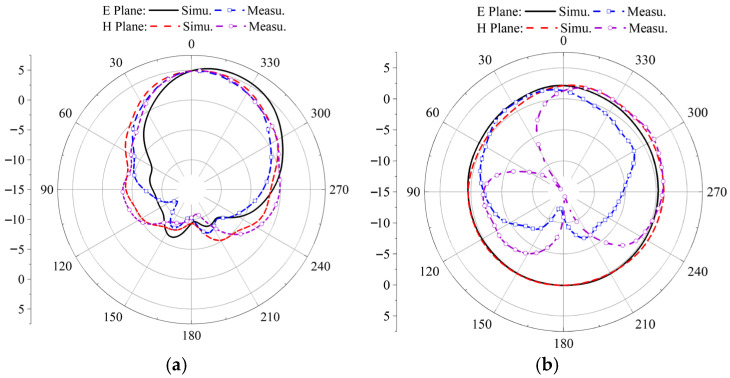
Simulation and measurement of radiation pattern, antenna at initial position: (**a**) 3.85 GHz, (**b**) 4.37 GHz.

The simulation results of the antenna’s S-parameters are in good agreement with the measured results. The antenna resonates at 3.92 GHz, with a relative impedance bandwidth of 48.17%. Subsequent tests were conducted in an anechoic chamber to evaluate the antenna’s axial ratio bandwidth, radiation gain, and radiation pattern. Figure 25 presents the antenna’s gain plot and axial ratio bandwidth plot. From Figure 25a, it can be observed that the simulated and measured gain results are close within the operating band, with an average gain of approximately 5.9 dBi. Figure 25b indicates that the antenna’s relative axial ratio bandwidth is 9.75%. Figure 26 shows the simulated and measured radiation patterns of the antenna at 3.85 GHz and 4.37 GHz, demonstrating that the measured results are in very good agreement with the simulations and that the antenna exhibits good directivity.

A sensitivity analysis was conducted to assess robustness. As shown in Figure 23, a rotation error of ±5° maintains the 3 dB AR bandwidth within acceptable limits (>8%). Similarly, substrate alignment offsets of up to 0.5 mm cause a gain variation of less than 0.8 dB and an XPD degradation of less than 3 dB, confirming the design’s tolerance to practical fabrication and assembly variances.

**Figure 27:**
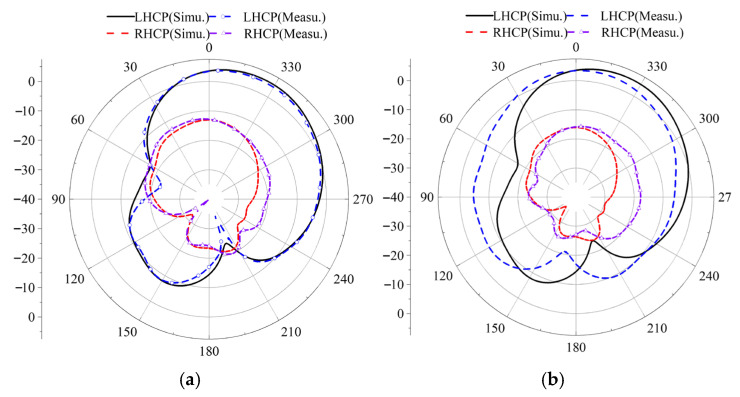
Simulation and measurement of the LHCP and RHCP at initial position: (**a**) phi = 0°, (**b**) phi = 90°.

At the initial position, the simulated and measured results of the antenna’s LHCP and RHCP are shown in Figure 27. In both the phi = 0° and phi = 90° planes, the LHCP component is approximately 21 dBi higher than the RHCP component, demonstrating excellent left-hand circular polarization characteristics.

**Figure 28:**
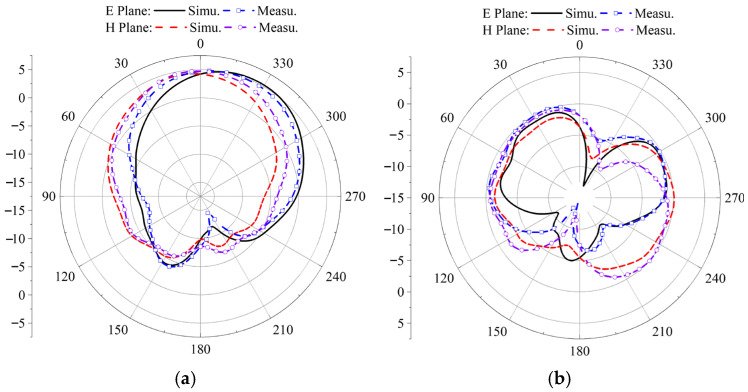
Simulation and measurement of antenna radiation patterns when rotating 90°: (**a**) 3.85 GHz, (**b**) 4.37 GHz.

**Figure 29:**
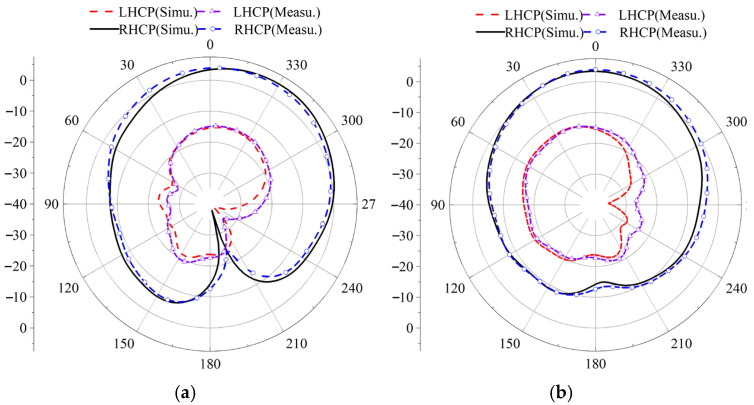
Simulation and test comparison of left-handed circular polarization and right-handed circular polarization at 90 ° rotation: (**a**) phi = 0°, (**b**) phi = 90° E plane.

After the antenna was rotated 90 degrees clockwise from its original configuration, a series of key performance measurements was conducted. These included the reflection coefficient |*S*_11_|, gain, axial ratio, and radiation patterns at two specific frequencies, 3.85 GHz and 4.37 GHz. The measured radiation patterns for both LHCP and RHCP in the two principal planes, phi = 0° and phi = 90°, are presented in Figure 28 and Figure 29, respectively. Both simulation and measurement results show strong agreement, confirming the antenna’s stable and satisfactory performance across these metrics.

It is noted that the operating band for the linear polarization (LP) state (≈4.3–5.9 GHz) is shifted higher compared to the circular polarization (CP) states (≈3.0–4.9 GHz). This shift arises from the change in the effective electrical dimensions and symmetry of the metasurface when rotated to 45°. Importantly, both bands reside within standard 5G sub-6 GHz allocations. The LP mode is particularly useful in scenarios where linear polarization offers superior discrimination against certain environmental clutter or interference patterns. A frequency-agile RF front-end can select the appropriate band and polarization state based on the operational context.

The use of FR4 substrate represents a cost-effective and readily fabricable choice for the prototype, validating the design concept. However, its relatively high dielectric loss tangent (tanδ = 0.02) at mid-GHz frequencies contributes to the measured ~80% radiation efficiency. For deployment in performance-critical UAV platforms, migrating the design to a low-loss laminate (e.g., Rogers RO4350B with tanδ ≈ 0.0037) is strongly recommended. Such a transition would boost the radiation efficiency to >90% with minimal design modification, at the expense of increased material cost. Environmental sealing (conformal coating) would also be advised for outdoor UAV operations to ensure stability against humidity and temperature variations.

A summary of the simulated and measured performance for the three polarization states is provided in Table 2. The data in the table indicates excellent consistency between simulated predictions and actual measured results. This close alignment not only validates the accuracy of the simulation model but also underscores the robustness and reliability of the antenna design in practical implementation [19].

### 4.2. Performance Analysis

**(i) Impedance Matching.** The antenna exhibits wide impedance bandwidths across all states. For the LHCP and RHCP states, the measured bandwidth exceeds 47%, which is remarkably wide. A slight frequency shift observed in the measurements, particularly for the LP state (e.g., measured −10 dB bandwidth is 29.9% vs. simulated 32.68%), is attributed to fabrication tolerances and the influence of the coaxial connector. The comparison between simulated and measured |*S*_11_| for the LHCP state demonstrates good agreement.

**(ii) Radiation Patterns.** The measured radiation patterns at representative frequencies (e.g., 3.85 GHz and 4.37 GHz) are stable and show good agreement with simulations.

**(iii) Gain and Polarization Purity.** The measured gain is stable across the operating bands, with an average of 5.7 dBi for LHCP and 5.0 dBi for RHCP. The measured axial ratio (AR) bandwidths are approximately 10.3% for LHCP and 10.1% for RHCP, confirming high-quality circular polarization. The LHCP-to-RHCP isolation exceeds 20 dBi in the boresight direction, indicating high polarization purity. Similarly, the LP state demonstrates a cross-polarization discrimination level greater than 23 dBi.

**(iv) Radiation Efficiency.** The measured radiation efficiency of the prototype was approximately 80% across the primary operating bands, with simulated efficiency exceeding 85%. The discrepancy is primarily attributed to the dielectric losses of the FR4 substrate (tanδ = 0.02) and minor losses from the coaxial connector. For high-performance drone applications where efficiency is paramount, the design can be directly transferred to lower-loss substrates (e.g., Rogers 4350B) with expected efficiency improvements above 90%.

Performance comparison with recent reconfigurable antennas is shown in Table 3. This work compares six other antenna designs from references [2,5,8,9,13,19]. The key parameters for comparison are size (in wavelengths, λ_0_), operating frequency, bandwidth (BW), gain, polarization (Pol.) states, reconfiguration method, and key features. Analysis and summary of the designed antenna’s advantages relative to other recent reconfigurable antennas are as follows.

The antenna boasts an exceptionally compact footprint of 0.49λ_0_ × 0.49λ_0_ × 0.07λ_0_. This size is smaller than several referenced designs (e.g., [2]: 0.76λ × 0.76λ, [8]: 0.57λ × 0.32λ, [13]: 0.39λ × 0.39λ) and is a critical advantage for integration into space-constrained drone terminals.

A primary innovation is the use of a mechanical switching (“Machine switch”) method. This is fundamentally different from the active methods (like PIN diodes in [2,9]) used in most other works. This passive approach eliminates insertion loss, DC power consumption, and design complexity associated with active electronic components, leading to higher reliability, which is crucial for drone applications.

The antenna can dynamically reconfigure among three states: left-hand circular polarization (LHCP), linear polarization (LP), and right-hand circular polarization (RHCP). Most other designs in the table offer only two states (e.g., RHCP/LHCP in [2,9,19]) or are limited to linear polarization [5]. This triple-state agility provides superior anti-jamming flexibility and channel adaptation capability for drone communication links.

Despite its compact size and passive mechanism, the antenna delivers strong, well-rounded performance:

Bandwidth: It achieves a measured 3 dB axial ratio (AR) bandwidth of 9.75% for circular polarization, which is a key metric for CP quality. While some references [5,8] claim much wider bandwidths, they often refer to different performance parameters (e.g., impedance BW for UWB) or different polarization states (LP only).

Gain: The average gain (5 dBi as listed, with detailed results showing 5.1 to 6.0 dBi) is stable across all three states and is comparable to or better than several other designs (e.g., 4.3 dBi in [2], ~5 dBi in [19]).

Polarization Purity: The design achieves high polarization purity, indicated by features like low axial ratio for CP states and high cross-polarization discrimination (>23 dB) for the LP state.

## 5. Conclusions

This paper has presented the design, fabrication, and measurement of a compact, polarization-reconfigurable metasurface antenna for sub-6 GHz drone terminals, systematically developed using characteristic mode analysis. The passive mechanical rotation mechanism enables reliable, low-loss switching among LHCP, LP, and RHCP states. The prototype demonstrates a wide impedance bandwidth (>29.9% for all states), stable gain, and high polarization purity, with excellent agreement between simulation and measurement.

The work also highlights inherent trade-offs and limitations. The millisecond-scale mechanical switching speed precludes real-time adaptive applications but is suitable for mission-phase-based reconfiguration. The bandwidth asymmetry between LP and CP states, while operationally manageable, points to an area for further structural optimization. Finally, the use of FR4 substrate, chosen for prototyping convenience, imposes an efficiency ceiling; future implementations should employ low-loss materials for optimal performance.

Future work will focus on (1) investigating fast, low-power actuation mechanisms (e.g., microelectromechanical systems) to reduce switching time; (2) exploring multilayer and multi-resonant metasurface designs to equalize and enhance bandwidth across all polarization states; and (3) integrating the antenna with sensing and control algorithms for autonomous polarization adaptation in dynamic channel environments.

## Figures and Tables

**Table 1:** Final size of the designed antenna.

**Dimension**	*w*_0_	*w*_1_	*w*_2_	*w*_3_	*w_r_*	*w_f_*
**Size** (mm)	11	3.68	2.5	2.5	35	2.0
**Dimension**	*L*	*l_f_*	*ls*	*h*_0_	*h*	*s*
**Size** (mm)	50	16.7	16	1.6	1.0	1.0

**Table 2:** Summary of antenna performance.

Polarization State	Parameter	Simulation	Measurement
**LHCP** (0°)	Impedance BW	48.17% (3.01–4.92 GHz)	47.0% (3.05–4.89 GHz)
	3 dB AR BW	9.75% (4.12–4.64 GHz)	10.3% (4.15–4.60 GHz)
	Avg. Gain	5.9 dBi	5.7 dBi
**LP** (45°)	Impedance BW	32.68% (4.30–5.98 GHz)	29.9% (4.33–5.85 GHz)
	XPD (Co/Cross-pol)	>23 dB	>23 dB
	Avg. Gain	5.3 dBi	5.1 dBi
**RHCP** (90°)	Impedance BW	48.29% (3.00–4.91 GHz)	48.5% (2.98–4.88 GHz)
	3 dB AR BW	9.98% (3.46–3.82 GHz)	10.1% (3.42–3.78 GHz)
	Avg. Gain	6.1 dBi	6.0 dBi

**Table 3:** Performance of the designed antenna compared to references.

Reference	Size (λ_0_)	Frequency (GHz)	BW (%)	Gain (dBi)	Pol. States	Reconfig. Method	Key Features
[2]	0.76 × 0.76 × 0.03	2.44–2.46	N/A	4.3	RHCP, LHCP	PIN diodes	Compact size
[5]	0.19 × 0.19 × 0.05	3.83–15.06	118.89	N/A	LP	1-bit phase modulation	UWB, Polarization rotation
[8]	0.57 × 0.32 × 0.013	2.45–14.88	~460	5.7	N/A	Orthogonal location	Compact size, UWB MIMO
[9]	0.2 × 0.2 × 0.042	2.45 5.8	1.8 2.3	0 1.5	RHCP, LHCP	Polarized filtering	Dual-Band, MIMO
[13]	0.39 × 0.39 × 0.09	4.98–6.00	18.6	6.1–7.5	Dual	Loading slots on the MTS	Metasurface antenna
[19]	N/A	4–5.7	29.8	5	LHCP, RHCP	Slot coupling	Compact size
**This Work**	**0.49 × 0.49 × 0.07**	**3.85–4.37**	**9.75**	**5**	**LHCP, LP, RHCP**	**Machine switch**	**Polarization stable**

## Data Availability

The original contributions presented in the study are included in the article, further inquiries can be directed to the corresponding author.
